# Coupling Carbon-Source Transport with Antioxidant Biosynthesis in Yeast: Product Diversity and Engineering Strategies

**DOI:** 10.3390/biology15181644

**Published:** 2026-09-17

**Authors:** Mao-Dong Zhang, Yu-Cheng Yao, Xin-Qing Zhao, Jun Li, Feng-Li Zhang

**Affiliations:** 1 State Key Laboratory of Microbial Metabolism, Joint International Research Laboratory of Metabolic & Developmental Sciences, School of Life Sciences and Biotechnology, Shanghai Jiao Tong University, Shanghai 200240, China; zmd0426@sjtu.edu.cn (M.-D.Z.); xqzhao@sjtu.edu.cn (X.-Q.Z.); 2 Shanghai CHANDO Group Co., Ltd., Shanghai 200233, China; 3 School of Biological Sciences, The University of Manchester, Oxford Rd., Manchester M13 9PL, UK; yucheng.yao@postgrad.manchester.ac.uk

**Keywords:** antioxidants, bioactive metabolites, biosynthesis, carbon-source transporters, yeast, yeast cell factories

## Abstract

Antioxidants protect cells from oxidative damage through mechanisms such as radical scavenging, metal chelation, and maintenance of redox homeostasis. Yeasts are useful hosts for antioxidant production. This review distinguishes native yeast metabolites, cell-wall and extracellular components, heterologously synthesized antioxidant compounds, fermentation- or extraction-associated antioxidants, and antioxidants obtained from mixed cultures. This review examines how carbon-source type and membrane transport influence central-carbon metabolism, precursor availability, and NADPH supply. These processes can affect antioxidant biosynthesis, although the outcome depends on the substrate, yeast strain, and biosynthesis pathway. Finally, we summarize engineering strategies involving transporter optimization, carbon-flux redistribution, enhancement of precursor and cofactor supply, and regulation of stress responses. Integrating carbon uptake with intracellular metabolic allocation provides a mechanistic framework for understanding and improving antioxidant production in yeast.

## Simple Summary

Yeasts are promising sources and production hosts for antioxidants used in food, health, and biotechnology, but not all compounds described as yeast-associated originate directly from yeast metabolism. This review summarizes the major classes and biological origins of yeast-associated antioxidants, distinguishing naturally produced metabolites and cell-wall or extracellular components from products generated through engineered yeasts, processing, fermentation, or mixed cultures. It then examines how yeasts take up sugars and other carbon sources and how these substrates influence energy generation, metabolic building blocks, and reducing power for antioxidant production. The review also discusses strategies involving transporter engineering, metabolic pathway optimization, product export, and fermentation-process control. By linking product origin with carbon uptake and production strategy, this review provides a framework for selecting suitable yeast hosts and feedstocks, and improving antioxidant production.

## 1. Introduction

Reactive oxygen species (ROS) are generated during aerobic metabolism and under environmental stress. When ROS formation exceeds cellular detoxification capacity, redox homeostasis is disrupted and lipids, proteins, and nucleic acids can be damaged [1,2,3]. Antioxidants mitigate these effects through mechanisms that include radical scavenging, metal chelation, and support of cellular redox systems [4]. Yeasts are promising production hosts because some species naturally accumulate antioxidant compounds, whereas others can be engineered to synthesize non-native products [5,6,7]. Antioxidant production nevertheless depends not only on pathway enzymes but also on carbon uptake and intracellular carbon allocation.

In this review, yeast-derived antioxidants are grouped into five categories: native intracellular antioxidants, cell-wall and extracellular components, heterologously synthesized compounds, fermentation- or extraction-associated products, and products obtained from mixed cultures. The first three categories are directly associated with yeast metabolism, whereas the latter two also involve raw-material transformation, extraction, or interactions among microorganisms in mixed cultures.

Carbon-source transport links extracellular substrates to intracellular antioxidant biosynthesis. After uptake, carbon is distributed through central metabolic pathways that supply energy, biosynthetic precursors, and reducing cofactors such as NADPH. Carbon- and redox-flux rebalancing improved β-carotene production in engineered *Yarrowia lipolytica* [8], while sucrose utilization and engineered malonyl-CoA supply increased heterologous resveratrol production in the same host [9]. Cofactor engineering likewise enhanced phenolic-acid production in *Saccharomyces cerevisiae* [10]. Previous reviews have generally discussed yeast antioxidants and sugar transport separately [6,7,11,12,13], leaving the relationship between transport and product metabolism insufficiently integrated. This review links antioxidant categories to carbon-source uptake and intracellular carbon allocation and summarizes strategies involving transporter optimization, carbon-flux redistribution, precursor and cofactor enhancement, and regulation of stress responses. The available evidence indicates that carbon transport can be a product- and substrate-dependent control point.

## 2. Antioxidants Associated with Yeast: Product Classes and Biological Origins

**Figure 1:**
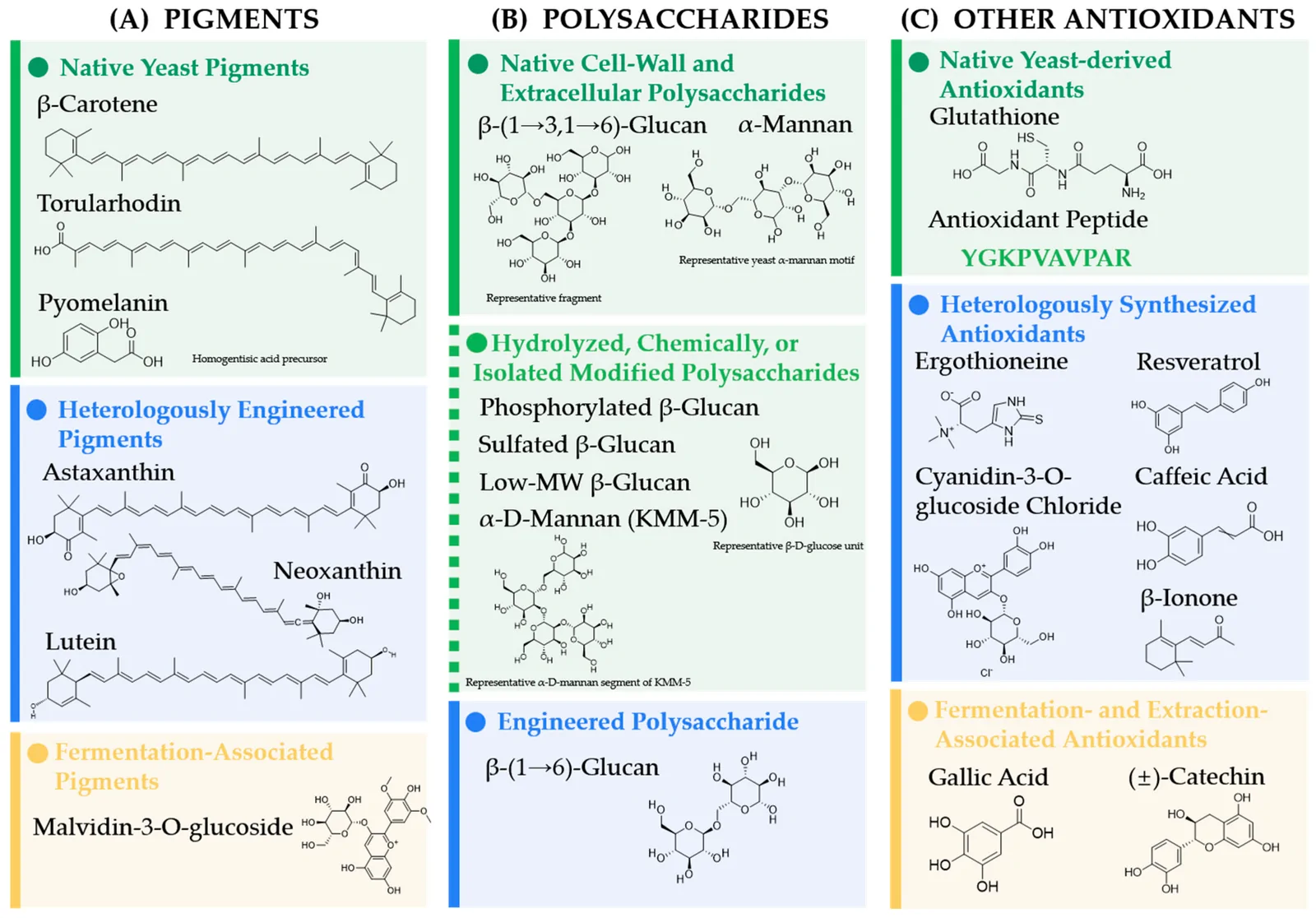
Classification of yeast-associated antioxidants by product class and biological origin. Representative products are grouped into three major classes: (**A**) antioxidant pigments; (**B**) polysaccharide antioxidants; and (**C**) phenolics, sulfur-containing compounds, peptides, and other antioxidants. The chemical structures illustrate representative compounds, constituent units, or oligosaccharide fragments; selected structural motifs are shown for heterogeneous polymeric products. Homogentisic acid is shown as the monomeric precursor of pyomelanin. Chemical structures were prepared using ChemDraw 23.1.1.3 (64-bit), and the figure was assembled in Microsoft PowerPoint.

Antioxidants inhibit or delay substrate oxidation when present at concentrations lower than those of the protected substrate [14]. Their effects can depend on concentration and chemical and biological conditions, and some compounds that exert antioxidant activity at low or moderate concentrations may become pro-oxidant at excessive concentrations [15]. In this review, yeast-associated antioxidants encompass compounds synthesized by yeast, cell-wall and extracellular components, products generated through heterologous pathway engineering, preparations obtained from yeast biomass or subsequent processing, and compounds associated with fermentation or mixed cultures. The yeast-associated antioxidants discussed here are first organized into three major classes—antioxidant pigments; polysaccharide antioxidants; and phenolics, sulfur-containing compounds, peptides, and other antioxidants. Based on their biological origins and the methods used for production and processing, these products are further classified as native, heterologously synthesized, isolated, hydrolyzed or chemically modified, biomass-derived, fermentation- or extraction-associated, and mixed-culture-associated products (Figure 1 and Table 1). Figure 1 and Table 1 summarize representative examples of yeast-associated antioxidants.

### 2.1. Antioxidant Pigments

#### 2.1.1. Native Yeast Pigments

Pigment antioxidants can be broadly produced in plant, microbial, and animal matrices. Frequently discussed examples include carotenoids, anthocyanins, chlorophylls and their derivatives, polyphenolic pigments, lutein, and melanins [16,17,18,19,20,21,22,23,24]. Hydroxyl, amino, thiol, and isoprenoid groups are recurrent structural motifs that can support radical scavenging, electron or hydrogen donation, metal binding, or quenching of reactive species, and many molecules contain more than one such motif [25]. These structural motifs help explain the interest in pigments for antioxidant, anti-inflammatory, food, cosmetic, and biomedical applications; however, occurrence in plants or animals does not establish a yeast biosynthetic origin.

Carotenoids are conjugated isoprenoid pigments whose extended polyene chains support quenching of reactive oxygen species and interruption of radical reactions [17,18,26,27]. Carotenogenic yeasts in the genera *Rhodotorula*, *Sporobolomyces*, *Sporidiobolus*, and *Xanthophyllomyces* naturally produce β-carotene, γ-carotene, torulene, and torularhodin [7,26,27,28]. Fed-batch cultures of *Sporidiobolus pararoseus* have produced mixtures of torularhodin, torulene, β-carotene, and γ-carotene, and antioxidant and related biological activities of these products have been evaluated [29,30]. Product composition varies with species, strain, carbon source, oxygen availability, and cultivation conditions. The measured concentrations of torularhodin and torulene also depend on extraction and purification procedures [18,28].

Melanins comprise chemically heterogeneous pigments commonly classified as eumelanins, pheomelanins, neuromelanins, allomelanins, and pyomelanins. Their functions include protection against ultraviolet and ionizing radiation, environmental stress tolerance, metal binding, pathogenesis, and cellular development [31,32,33,34,35]. Fungal melanization can proceed through L-DOPA-, 1,8-dihydroxynaphthalene-, or homogentisic-acid-dependent routes. Native melanizing yeasts and yeast-like fungi include *Y. lipolytica*, *Exophiala dermatitidis*, and *Cryptococcus rajasthanensis* [24,35,36]. Pyomelanin from *Y. lipolytica* has been characterized as non-cytotoxic and as having antioxidant and ultraviolet-protective activity, while melanin isolated from *C. rajasthanensis* KY627764 showed antioxidant, antibacterial, and photoprotective activities [24,37]. Laccase-mediated production of non-cytotoxic pyomelanin has also been compared with bacterial and synthetic pigments [38]. These studies illustrate both the native diversity of fungal pigments and the analytical difficulty poseed by melanin’s heterogeneous polymeric structure. A complementary example is the black yeast-like fungus *Exophiala mesophila*, for which inhibitor experiments and spectroscopic analyses support predominantly 1,8-dihydroxynaphthalene-derived melanin [39].

#### 2.1.2. Heterologously Engineered Pigments

Engineering yeasts for pigment production has advanced rapidly. Recent work on β-carotene derivatives from *Saccharomyces cerevisiae* and *Y. lipolytica* encompasses oxygenated xanthophylls—canthaxanthin, zeaxanthin, astaxanthin, violaxanthin, lutein, and neoxanthin—and apocarotenoids such as crocetin, retinol, β-ionone, β-cyclocitral, and strigolactones [40]. Lutein production in engineered *S. cerevisiae* required temporospatial control of the introduced pathway [41]. Astaxanthin production was enabled by integrating and tuning algal β-carotene hydroxylase and ketolase genes, while additional pathway combinations produced canthaxanthin and zeaxanthin [42,43]. A heterologous xanthophyll pathway, balanced precursor supply, and adjustment of the membrane-related metabolic environment enabled neoxanthin biosynthesis [44]. In food-grade *Candida utilis*, introduction of a bacterial pathway enabled lycopene, β-carotene, and astaxanthin production [45]; in *Y. lipolytica*, carbon-flux redistribution and redox rebalancing improved β-carotene titer and yield [8].

Melanin can be obtained through native pathway amplification or engineered production. *Y. lipolytica* possesses an intrinsic homogentisic-acid pathway; enhancing aromatic amino acid precursor supply and increasing 4-hydroxyphenylpyruvate dioxygenase gene dosage increased the pyomelanin titer to 4.5 g/L [36]. In contrast, methanol-based pyomelanin production in engineered *Komagataella phaffii* is an introduced production system and should not be generalized as a native yeast trait [46]. Porphyrins are also relevant as tetrapyrrole scaffolds shared with heme and chlorophyll-related chemistry. Heme-supply engineering has been demonstrated in *K. phaffii* (formerly *Pichia pastoris*), and synthetic-biology strategies for porphyrin production in yeast have been reviewed [47,48]. These studies support production of porphyrins or heme-related products, but chlorophyll biosynthesis by yeast has not been demonstrated.

#### 2.1.3. Fermentation and Mixed-Culture-Associated Pigments

Anthocyanins and anthocyanin-derived products are prominent in yeast-fermented beverages [49]. Anthocyanidins share a flavylium-based C_6_-C_3_-C_6_ scaffold with hydroxyl and methoxyl substitutions that influence color and reactivity [50,51]. During beverage fermentation, yeast can transform, adsorb, release, or stabilize anthocyanins and can alter their accessibility through changes in ethanol, pH, and matrix composition. Nevertheless, the carbon skeletons of beverage anthocyanins generally originate from plant raw materials; therefore, anthocyanins are fermentation-associated products rather than native yeast pigments. This category is distinct from de novo anthocyanin biosynthesis in strains carrying introduced plant genes [52,53,54].

The redox and radical-quenching properties of chlorophylls and their derivatives have been examined extensively [20,22]. Chlorophyll biosynthesis has not been established in the yeast hosts discussed here. Reports that yeast addition or glycerol supplementation increases chlorophyll in a yeast–alga culture refer to chlorophyll produced by the algal partner, such as *Chlorella vulgaris*, rather than by yeast [55]. Accordingly, changes in chlorophyll levels in yeast–alga cocultures should be attributed to the algal partner unless direct evidence demonstrates yeast-derived chlorophyll biosynthesis.

### 2.2. Polysaccharide Antioxidants

#### 2.2.1. Native Cell-Wall and Extracellular Polysaccharides

Yeast exopolysaccharides are high-molecular-weight glycosidic polymers secreted by genera including *Bullera*, *Candida*, *Cryptococcus*, *Debaryomyces*, *Lipomyces*, *Pichia*, *Pseudozyma*, *Rhodotorula*, and *Sporobolomyces*. They are commonly mannose- and glucose-rich heteropolysaccharides with branched α-mannose or β-glucose backbones and strain-dependent side-chain linkages, molecular masses, and phosphate, sulfate, or acetyl substitutions [56]. Culture composition and extraction methods affect the recovered structure and measured activity. Mannose- and glucose-containing exopolysaccharides from *Debaryomyces hansenii* DH-1 showed antioxidant and antitumor activities, and a sulfated fraction isolated from *S. cerevisiae* HD-01 showed reducing and radical-scavenging activity [57,58].

β-Glucans and mannans are major structural components of the yeast cell wall rather than conventional intracellular secondary metabolites. Yeast β-glucans consist mainly of β-1,3-linked glucose backbones with variable β-1,6 branching, whereas mannans contain mannose-rich backbones and side chains whose linkage patterns and abundance vary among species and strains [59,60,61,62,63,64]. These polymers contribute to cell wall integrity and host–microbe interactions and have been investigated for immunomodulatory, antimicrobial, prebiotic, and antioxidant properties. Their biosynthesis connects directly to central-carbon metabolism through activated sugar donors, particularly UDP-glucose for glucans and GDP-mannose for mannans.

#### 2.2.2. Isolated, Hydrolyzed, and Chemically Modified Polysaccharides

Most reports on antioxidant activity of yeast cell-wall polysaccharides concern isolated, hydrolyzed, or chemically modified preparations rather than intact yeast cells. Molecular weight, branching, solubility, concentration, and functional-group substitution strongly influence the bioactivity [60,65]. Alkali-insoluble (1 → 3)-β-D-glucan and six derivatives—sulfated, carboxymethylated, phosphorylated, carboxymethylated-phosphorylated, carboxymethylated-sulfated, and sulfated-phosphorylated glucans—have been compared directly. Sulfated material exhibited substantial reducing capacity, whereas phosphorylated derivatives showed hydroxyl-radical and superoxide-anion scavenging and inhibition of lipid peroxidation [66,67]. In mice, phosphorylated (1 → 3)-β-D-glucan increased superoxide dismutase and catalase activities in serum, liver, and brain and reduced malondialdehyde concentrations under the tested conditions [68]. These results should be interpreted as specific to the tested preparations and assays rather than as evidence of equivalent activity in unprocessed biomass.

Mannan fractions show size- and concentration-dependent activities. Mannans of varying molecular weights isolated from *S. cerevisiae* cell walls scavenged DPPH radicals [69]. A 203 kDa of α-D-mannan (KMM-5) and related cell-wall polysaccharides from *Kluyveromyces marxianus* CCT7735 displayed copper- and iron-chelating capacity, while four additional fractions demonstrated total antioxidant activity [70,71]. Mannan extracts from *S. cerevisiae* were evaluated as prebiotic ingredients in bio-yogurt systems [72] and as antioxidant and growth-promoting substrates for *Lactobacillus* strains [73].

#### 2.2.3. Engineered and Mixed-Culture Production

Polysaccharide engineering can focus on polymer synthesis as well as carbon supply. Heterologous expression of the β-1,6-glucan synthase GsmA from *Mycoplasma agalactiae*, enhancement of endogenous glucan-synthesis reactions, and fermentation optimization increased glucan biosynthesis in *S. cerevisiae* [74]. This work represents engineered accumulation of the native cell-wall polysaccharide class rather than introduction of an unrelated antioxidant pathway.

Mixed-culture polysaccharide studies require specific organism attribution. Co-culturing the bacterium *Weissella confusa* XG-3 with the yeast *Candida shehatae* increased bacterial exopolysaccharide production to 115.66 g/L, approximately 2.04-fold higher than the reported monoculture value [75]. This case is relevant to yeast-assisted process intensification, but the polymer is from *W*. *confusa*, not yeast.

### 2.3. Phenolics, Sulfur-Containing Compounds, Peptides, and Other Antioxidants

#### 2.3.1. Native Intracellular and Biomass-Derived Products

Glutathione is a central intracellular thiol antioxidant in yeast and can also be obtained from enriched biomass. Selenium/glutathione-enriched *C. utilis* was prepared and evaluated for biological effects in rats [76]. Ergothioneine is another sulfur-containing antioxidant of biotechnological interest; high-level production in *Y. lipolytica* was achieved by strengthening precursor supply, optimizing sulfur-related metabolism, using enzyme fusion, and integrating multiple pathway copies [77]. The latter is an engineering outcome and should not be treated as evidence that ergothioneine is a universally abundant native yeast metabolite.

Antioxidant peptides from *S. cerevisiae*, *K. marxianus*, and *Saccharomyces pastorianus* are typically released by enzymatic hydrolysis of yeast proteins rather than secreted as final antioxidant products during fermentation [78,79,80]. Reported peptides commonly contain 3–20 residues and are enriched in combinations of His, Tyr, Cys, Trp, or Met together with hydrophobic residues such as Leu, Val, and Phe. Sequence, molecular size, hydrophobicity, hydrolysis conditions, and purification determine radical scavenging activity, metal chelation, reducing capacity, solubility, and membrane interaction. These preparations are classified as biomass-derived products.

#### 2.3.2. Heterologously Synthesized Phenolics

Phenolic compound families discussed in yeast-related studies include flavonoids, tannins, phthalans, xanthones, phenolic acids, stilbenoids, and related derivatives [81,82]. However, the evidence for their biosynthesis by yeast differs substantially among these classes. Flavonoids possess a C_6_-C_3_-C_6_ skeleton comprising two aromatic rings linked by a three-carbon unit [83,84]. Phenolic metabolites from an engineered flavonoid-producing *S. cerevisiae* strain exhibited scavenging activity against the radical cation of N,N-dimethyl-p-phenylenediamine (DMPD^+^) [85]. Six flavonoids—naringenin, liquiritigenin, kaempferol, resokaempferol, quercetin, and fisetin—were produced from glucose in engineered yeast [86].

Complete heterologous pathways have enabled anthocyanin production in *S. cerevisiae* through introduction of plant-derived enzymes, pathway balancing, organelle or membrane engineering, expression of the glutathione S-transferase MdGSTF6 and reduction of endogenous anthocyanin degradation [52,53,54]. Phenolic-acid production, including caffeic- and ferulic-acid routes, was improved by engineering the supply and recycling of FADH_2_, *S*-adenosyl-L-methionine, and NADPH [10]. Resveratrol and related stilbenoids were produced by heterologous expressions in *S. cerevisiae* and *Y. lipolytica* [9,87,88]. These pathways connect carbon uptake to phosphoenolpyruvate, erythrose 4-phosphate, aromatic amino acid intermediates, malonyl-CoA, and redox-cofactor supply.

Tannins have additional complexity arising from polymerization and stereoisomerism, and no convincing report has yet demonstrated complete de novo synthesis of structurally diverse tannin molecules in yeast. Likewise, conventional yeast does not naturally synthesize xanthones, and complete de novo yeast production remains insufficiently established. Therefore, tannins and xanthones should not currently be classified as established yeast products without direct pathway and product-identification evidence.

#### 2.3.3. Fermentation-, Extraction-, and Mixed-Culture-Associated Products

Phenolic compounds detected in yeast-fermented or yeast-derived preparations are not necessarily synthesized de novo through yeast carbon metabolism. Yeast may release matrix-bound phenolics, transform glycosides or side chains, modify solvent properties, adsorb compounds to the cell surface, or alter their bioaccessibility [81]. Gallic acid and (±)-catechin quantified in lyophilized brewer’s spent yeast extracts can include compounds originating from malt and hops that remain associated with biomass after brewing [89]. The same attribution principle applies to raw-material phenolics in foods and to metabolites recovered from yeast–bacterium communities: fermentation association is not equivalent to de novo yeast biosynthesis. Methanol and acetone extracts of the Antarctic isolates *Dioszegia* sp. AL105 and *Bannozyma* sp. AL104 were evaluated for antioxidant and cytotoxic activities [90]. Such measurements characterize the tested extracts and should not be attributed to a single pigment without compound-specific evidence.

### 2.4. Comparative Overview and Metabolic Implications

Yeasts offer practical advantages as production hosts, including growth at relatively low pH, genetic tractability, eukaryotic protein-processing capacity, and suitability of selected strains for food and nutraceutical applications [11,91,92,93]. Native and heterologous intracellular antioxidants depend directly on precursor and cofactor supply. Cell-wall and extracellular polysaccharides depend on sugar-nucleotide formation, polymerization, and secretion, whereas biomass-derived, extraction-associated, and mixed-culture products are related to downstream processing, raw materials, and co-cultured organisms. Host selection also depends on the available expression tools, protein processing, secretion capacity, and substrate utilization, which differ among conventional and non-conventional yeasts [94,95].

Accordingly, carbon sources cannot influence every antioxidant category through the same mechanism. For native or heterologous intracellular products, transport can affect central-carbon flux, acetyl-CoA, aromatic precursors, malonyl-CoA, and NADPH. For cell-wall polysaccharides, the relevant link is activated-sugar supply and polymer assembly. For beverage, extraction, or mixed-culture products, yeast metabolism may alter recovery or transformation without supplying the product’s complete carbon skeleton. These distinctions provide the basis for examining carbon transport as a product- and substrate-dependent control point in the following section.

**Table 1:** Classification of yeast-associated antioxidants by product class and biological origin.

Product Class	Biological Origin			References
	Native Yeasts	Engineered Yeasts	Fermentation-, Extraction-, or Mixed-Culture Yeasts with Other Microbes	
Pigments	Carotenoids; melanins	Carotenoids and xanthophylls; pyomelanin	Beverage anthocyanins; algal chlorophyll in coculture	[8,24,26,27,28,36,37,40,41,42,43,44,45,46,49,55]
Polysaccharides	β-glucans and mannans; exopolysaccharides	Glucan	Mixed-culture-associated polysaccharides	[56,57,58,59,60,61,62,63,66,67,68,69,70,71,73,74,75,96]
Phenolics	Not established in the examples reviewed	Flavonoids; anthocyanins; phenolic acids; stilbenoids	Raw-material phenolics; fermentation-transformed or recovered phenolics	[9,10,49,52,53,54,81,85,86,87,88,89]
Sulfur-containing compounds and peptides	Glutathione	Ergothioneine	Protein-hydrolysate peptides; processing-recovered metabolites	[76,77,78,79,80]

## 3. Coupling Carbon-Source Transport with Antioxidant Biosynthesis in Yeast

Carbon-source transport links the extracellular environment to intracellular metabolism. Yeasts can assimilate soluble sugars, glycerol, organic acids, and fatty acids, whereas complex polymers must first be converted into transportable molecules. After uptake, these substrates enter central metabolism, contributing to energy generation, precursor formation, and redox-cofactor supply. Therefore, transport capacity can influence antioxidant production, but its importance depends on the substrate, transporter, yeast strain, and product pathway.

### 3.1. General Mechanisms of Carbon-Source Uptake

Many soluble sugars cross the yeast plasma membrane via facilitated diffusion or proton-coupled symport [12,13,97]. Facilitated diffusion moves substrates down their concentration gradients without direct energy expenditure, whereas proton symport can support uptake at low extracellular substrate concentrations. Transporter affinity, specificity, abundance, localization, and regulation collectively determine uptake capacity. Transport should not be assumed to be rate limiting because pathway enzymes, precursor formation, cofactor supply, and product toxicity also play roles in a given production system.

### 3.2. Transport of Representative Carbon Sources

#### 3.2.1. Hexoses and Pentoses

Sugars are preferred carbon sources for many yeasts, and plasma-membrane transport is an initial step in sugar assimilation. Facilitated-diffusion transporters move substrates down concentration gradients without direct energy expenditure, whereas proton-coupled symporters can concentrate sugars when extracellular concentrations are low [12,13,97]. In *S. cerevisiae*, the Hxt network comprises Hxt1–Hxt17 and Gal2, which differ in affinity, capacity, expression pattern, and substrate range. Blocking hexose uptake requires deletion of many paralogous transporters, illustrating the redundancy of the system [98]. Snf3 and Rgt2 are homologous membrane glucose sensors that regulated transporter expression but do not function as ordinary glucose carriers; therefore they should be separated from the Hxt/Gal2 transporters in mechanistic descriptions [99].

Transporters vary substantially among non-model yeasts. Hgt1 mediated high-affinity glucose uptake in *Kluyveromyces lactis*, whereas a family of Yht proteins contributes to glucose, fructose, and mannose utilization in *Y. lipolytica* [100,101]. In *Torulaspora delbrueckii*, Lgt1 was a low-affinity glucose transporter and Igt1 was a dual-affinity carrier that transported glucose, fructose, and mannose [102,103]. Fructose-specific or fructose-preferring systems include Ffz1 in *Zygosaccharomyces bailii*, Frt1 in *K. lactis*, and Fsy1-type proton symporters found in several *Saccharomyces* species and related yeasts [104,105,106]. These cases illustrate why transporter names should not be transferred between species without functional evidence.

Pentose utilization is often limited by low transporter affinity, competition with glucose, and carbon catabolite repression. Native Hxt and Gal2 proteins weakly transport xylose or L-arabinose, and engineering Gal2 improved L-arabinose transport in *S. cerevisiae* [107,108]. In *Candida intermedia*, Gxf1 primarily functions as a facilitated glucose/xylose transporter, whereas Gxs1 was a glucose/xylose–H^+^ symporter [109]. Axt proteins from *K. marxianus* and *Meyerozyma guilliermondii* (formerly *Pichia guilliermondii*) enabled growth on L-arabinose and D-xylose when expressed in *S. cerevisiae* [110]. AraT from *Scheffersomyces stipitis* improved L-arabinose uptake, while Sut1–Sut3 from the same species are broad-specificity glucose transporters with pentose activity [111,112]. D-arabinose transporters were characterized in *S. stipitis* [113].

The *Candida sojae* transporter Cs4130 has also been functionally tested in a hexose-transporter-deficient, xylose-utilizing *S. cerevisiae* strain. Heterologous expression supported growth and sugar consumption, and fluorescence imaging showed plasma-membrane localization. At 50 g/L of xylose, the Cs4130-expressing strain consumed xylose approximately 30% faster than the Gxf1-expressing control over 24 h; glucose nevertheless inhibited xylose utilization in mixed-sugar cultures [114]. These results support substrate-dependent transporter selection, but do not establish an improvement in antioxidant production.

Additional pentose systems expand the available engineering tools. In a modular yeast consortium, expression of SpXut1 from *Spathaspora passalidarum* in *Scheffersomyces stipitis*, together with engineering of xylose assimilation, improved mixed-sugar utilization and shikimate production [115]. *Spathaspora passalidarum* likewise provided transporter and metabolic resources for pentose utilization [116]. However, transporter function, intracellular metabolism, and redox balance must all be adequate for improved product formation. Glucose can repress or competitively inhibit transport of xylose and arabinose, so a carrier that performs well in single-sugar medium may not improve mixed-sugar conversion. Transporter choice should therefore be evaluated using the actual feedstock composition and linked to downstream precursor and cofactor demands rather than inferred from uptake alone [117,118].

#### 3.2.2. Disaccharides

Yeasts utilize sucrose either by extracellular hydrolysis or by direct uptake. In the first route, invertase releases glucose and fructose, which enter via monosaccharide transporters; in the second, a sucrose–H^+^ symporter imports intact sucrose for intracellular hydrolysis [119,120]. Members of the *Wickerhamiella*/*Starmerella* clade used distinct combinations of these strategies, and heterologous sucrose transport enabled direct sucrose conversion in engineered *S. cerevisiae* [119,120]. The two routes differ in energy demand, extracellular sugar accumulation, and competition for hexose transporters.

Lactose utilization requires β-galactosidase together with a compatible uptake system when lactose hydrolysis occurs intracellularly. Lac12 mediates lactose and galactose transport in *K. lactis*, and its localization is regulated by carbon-signaling pathways [121]. Maltose and maltotriose are imported by Malx1-, Agt1-, Mtt1-, and Mph-family transporters in *Saccharomyces* yeasts; transporter complements help explain strain-specific performance in brewing and starch-derived media [122,123,124,125]. Group I *S. pastorianus* strains and lager-type *AGT1* alleles illustrate how transporter sequence and copy number affect maltose or maltotriose utilization. Simultaneous glucose and maltose uptake can also impair growth in *S. cerevisiae*, showing that broader substrate access is not automatically beneficial unless regulatory and metabolic loads are balanced [122].

#### 3.2.3. Glycerol, Organic Acids, and Fatty Acids

Glycerol is an inexpensive reduced carbon source, but its uptake is condition-dependent. Fps1 is an aquaglyceroporin that facilitates glycerol movement across the membrane, particularly during osmotic adaptation, whereas Stl1 is the principal proton-coupled active glycerol importer in *S. cerevisiae* under glycerol-utilizing conditions [126,127,128,129]. Gup1 and Gup2 were historically described as glycerol-uptake components, but subsequent work indicates broader roles in membrane and lipid biology; they should not be presented as unambiguous glycerol transporters [127]. Functional studies in *Pachysolen tannophilus* and adaptive evolution of *S. cerevisiae* have further showed that glycerol growth depends on both transport and intracellular catabolic capacity [129,130].

Carboxylic-acid uptake depends on acid pK_a_, extracellular pH, and membrane transport. At pH values below the pK_a_, the undissociated form can cross the membrane by diffusion; at higher pH, anionic species depend more strongly on carriers [131,132]. Jen1 transports lactate, pyruvate, and related monocarboxylates in *S. cerevisiae*, Ady2 is important for acetate uptake, and Mae1 mediates malate and other C4-dicarboxylate transport in *Schizosaccharomyces pombe* [133,134]. Fps1 can also facilitate entry of undissociated acetic acid under particular conditions, linking its channel activity to acid sensitivity [126]. These multiple routes make pH control as important as transporter abundance.

Long-chain fatty-acid assimilation involves membrane partitioning, protein-assisted uptake, activation to acyl-CoA, and delivery to peroxisomes for β-oxidation. In *S. cerevisiae*, Fat1 contributes to fatty-acid transport and very-long-chain acyl-CoA synthetase activity, while the Pxa1/Pxa2 half-ABC transporter complex imports activated substrates into peroxisomes [135,136]. In *Y. lipolytica*, acyl-CoA synthetases supported fatty-acid and *n*-alkane utilization [137]. β-Oxidation supplies acetyl-CoA for the TCA or glyoxylate cycle and can therefore feed mevalonate-derived carotenoids. However, transporter changes alone will not increase production if activation, peroxisomal flux, or NADPH supply remains limiting. Representative transporters involved in the uptake of carbon sources and related substrates in yeast species are shown in Table 2.

**Table 2:** Representative transporters involved in the uptake of carbon sources and related substrates in yeast species.

Substrate Class/Group		Substrate	Transporter or Uptake-Related Protein	Yeast Species	References
Monosaccharide	Pentose	Xylose	Axt1	*K. marxianus* or *P. guilliermondii*	[110]
			Gal2	*S. cerevisiae*	[13]
			Gxf1	*C. intermedia*	[109]
			Gxs1	*C. intermedia*	[109]
			Hxt family	*S. cerevisiae*	[13]
			Sut1–3	*S. stipitis*	[13,111]
			SpXut1	*Spathaspora passalidarum*	[115]
			Cs4130	*Candida sojae*	[114]
		L-Arabinose	AraT	*S. stipitis*	[112]
			Axt1	*K. marxianus* or *P. guilliermondii*	[110]
			Gal2	*S. cerevisiae*	[107]
			Hxt9/Hxt10	*S. cerevisiae*	[13,112]
	Hexose	Glucose	Gal2	*S. cerevisiae*	[13]
			Gxf1	*C. intermedia*	[109]
			Gxs1	*C. intermedia*	[109]
			Hgt1	*K. lactis*	[101]
			Hxt family	*S. cerevisiae*	[13,98]
			Igt1	*Torulaspora delbrueckii*	[103]
			Lgt1	*T. delbrueckii*	[102]
			Sut1–3	*S. stipitis*	[111]
			Yht family	*Y. lipolytica*	[100]
		Galactose	Gal2	*S. cerevisiae*	[13,107]
			Hxt14	*S. cerevisiae*	[13,98]
			Yht family	*Y. lipolytica*	[100]
		Fructose	Ffz1	*Zygosaccharomyces bailii*	[105]
			Frt1	*K. lactis*	[106]
			Fsy1	*S. bayanus* and *S. pastorianus*	[104]
			Hxt family	*S. cerevisiae*	[13,98]
			Igt1	*T. delbrueckii*	[103]
			Lgt1	*T. delbrueckii*	[102]
			Yht family	*Y. lipolytica*	[100]
Disaccharide		Sucrose	Sucrose permease(s)	*Wickerhamiella* and *Starmerella* spp.	[119]
		Lactose	Lac12	*K. lactis*	[121]
		Maltose	Agt1	*S. pastorianus*	[125]
			Malx1 family	*S. cerevisiae*	[123]
			Mph2/Mph3	*S. cerevisiae*	[123]
			Mtt1	*S. pastorianus*	[123]
Organic acids	Carboxylic acids	Monocarboxylates	Ady2	*S. cerevisiae*	[134]
			Fps1	*S. cerevisiae*	[126]
			Jen1	*S. cerevisiae* and other yeasts	[131,132]
		Fatty acids/fatty acyl-CoA	Fat1	*S. cerevisiae*	[135]
			Faa1 family (acyl-CoA synthetases)	*Y. lipolytica*	[137]
			Pxa1/Pxa2	*S. cerevisiae*	[136]
Glycerol			Fps1	*S. cerevisiae*	[127]
			PtFps2	*Pachysolen tannophilus*	[129]
			Stl1	*S. cerevisiae* and *S. kudriavzevii*	[127,128]

Transporter names are presented according to the nomenclature used in the cited studies. Yeast species names are abbreviated after their first occurrence where applicable.

#### 3.2.4. Complex and Waste-Derived Feedstocks

Complex polymers are not plasma-membrane substrates. Native *S. cerevisiae* does not efficiently use raw starch or inulin because extracellular depolymerization is limiting; raw-starch conversion requires secreted or surface-displayed amylolytic enzymes, and inulin utilization depends on inulinase activity before the released sugars are transported [138,139]. Wild-type *K. marxianus* strains can use sucrose, raffinose, and inulin, and disruption of carbon repression together with inulinase overexpression has improved inulinase production [139,140]. These cases involve extracellular enzyme access and regulatory control rather than direct transport of intact polysaccharides.

**Figure 2:**
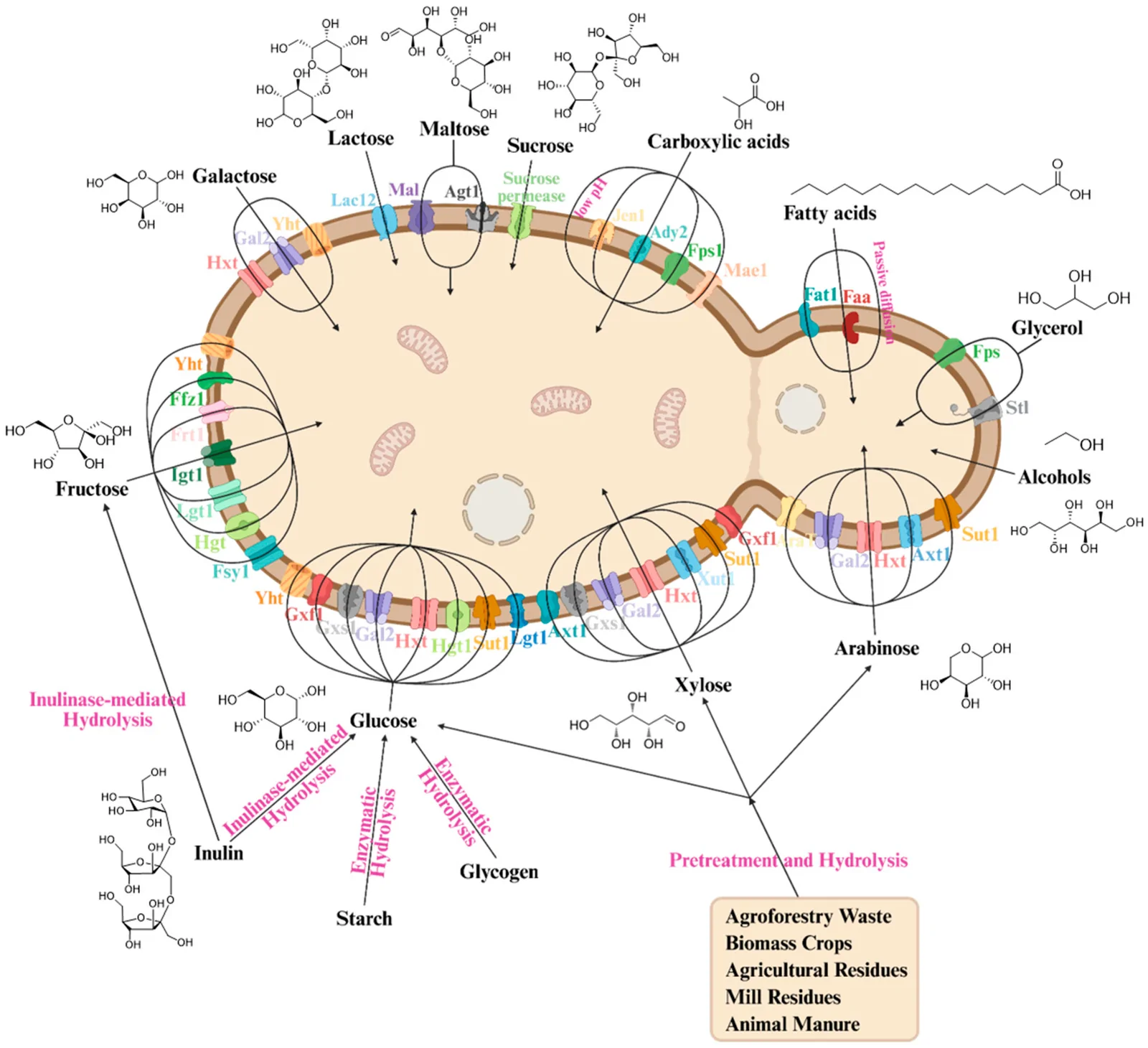
Representative carbon sources and routes of substrate acquisition in yeast. The schematic summarizes transport systems associated with the uptake of monosaccharides, disaccharides, glycerol, and organic acids, together with fatty-acid uptake and activation. Pretreatment and/or extracellular hydrolysis release soluble substrates from polymeric and waste-derived feedstocks before cellular uptake, including inulinase-mediated hydrolysis of inulin. Passive diffusion provides an additional entry route for certain substrates under suitable conditions. Fatty-acid uptake can be coupled to intracellular activation to acyl-CoA, as illustrated by the cooperation between Fat1 and the fatty acyl-CoA synthetases Faa1/Faa4 in *Saccharomyces cerevisiae*. The illustrated systems represent examples from different yeast species and are not necessarily present together in a single strain. Arrows indicate representative substrate-release and uptake routes rather than relative transport rates or metabolic fluxes. Transporter identities, source species, substrate ranges, and supporting references are summarized in Table 2. The figure was prepared using BioRender.com and Microsoft PowerPoint.

Agricultural residues, forestry wastes, energy crops, mill residues, and other biomass streams contain cellulose, hemicellulose, lignin, and variable amounts of soluble sugars, organic acids, lipids, and inhibitors [141,142,143]. Pretreatment and enzymatic hydrolysis release glucose, xylose, and arabinose, after which the monosaccharide transporters described above become relevant [144,145]. Feedstock performance therefore reflects a sequence of operations—pretreatment, hydrolysis, inhibitor tolerance, substrate transport, central-carbon metabolism, and product formation. Efficient utilization of waste-derived feedstocks therefore requires coordinated improvement of substrate release, inhibitor tolerance, uptake, and metabolic conversion. Non-conventional yeasts can use diverse organic waste streams, but their suitability depends on feedstock composition, inhibitory compounds, and the metabolic capabilities of the selected host [146]. Representative carbon sources and routes of substrate acquisition in yeast are shown in Figure 2.

### 3.3. Links Between Carbon Uptake and Antioxidant Biosynthesis

Carbon uptake influences antioxidant production by directing imported carbon toward energy generation, precursor formation, and cofactor regeneration. Carbon-source comparisons and precursor or cofactor engineering establish links between substrate metabolism and product formation, but do not by themselves identify membrane transport as the controlling step. Establishing a transporter-specific contribution requires defined transporter perturbations together with measurements of substrate uptake and product formation under matched cultivation conditions, while accounting for changes in growth and downstream metabolism.

For carotenoids, the principal metabolic requirements include acetyl-CoA-derived isoprenoid precursors and NADPH. In engineered *Y. lipolytica*, coordinated carbon- and redox-flux rebalancing produced 809.2 mg/L β-carotene at a yield of 0.04 g/g glucose in shake-flask cultivation [8]. In engineered *S. cerevisiae*, β-carotene production was threefold higher on xylose than on glucose, and subsequent xylose-fed-batch cultivation reached 772.8 mg/L. The authors associated the advantage of xylose with a more respiratory metabolic state and greater cytosolic acetyl-CoA availability, rather than demonstrating an isolated effect of transporter engineering [147].

Heterologous phenolic pathways depend on distinct combinations of precursors and cofactors, including aromatic amino acid intermediates and, for some products, malonyl-CoA. In *Y. lipolytica* grown in glucose-containing medium, combined engineering of malonyl-CoA-forming modules and malonate uptake, together with malonate supplementation, increased resveratrol titers from 14.3 to 51.8 mg/L [9]. In *S. cerevisiae*, systematic cofactor engineering enabled caffeic acid and ferulic acid titers of 5.5 ± 0.2 g/L and 3.8 ± 0.3 g/L, respectively [10]. These outcomes illustrate how intracellular precursor availability and cofactor balance can constrain product formation even when the carbon substrate is accessible.

Cell-wall polysaccharide production depends on activated sugar donors, including UDP-glucose for β-glucans and GDP-mannose for mannans [63,96]. Consequently, additional carbon uptake may provide limited benefit when sugar-nucleotide formation or polymerization is limiting [74]. For sulfur-containing antioxidants, carbon metabolism supports amino acid precursor formation and ATP generation, whereas selenium/glutathione-enriched biomass [76] and engineered ergothioneine production [77] represent different product forms. Fermentation-associated and mixed-culture preparations additionally depend on raw-material transformation and interactions among microorganisms.

The relationship between carbon uptake and antioxidant production therefore depends on the intracellular fate of the imported substrate and the requirements of the target product. Uptake measurements should be interpreted alongside product yield, productivity, biomass formation, and by-product accumulation to identify the relevant control points. Representative production studies are summarized in Table 3.

### 3.4. Engineering Implications and Evidence Limitations

**Figure 3:**
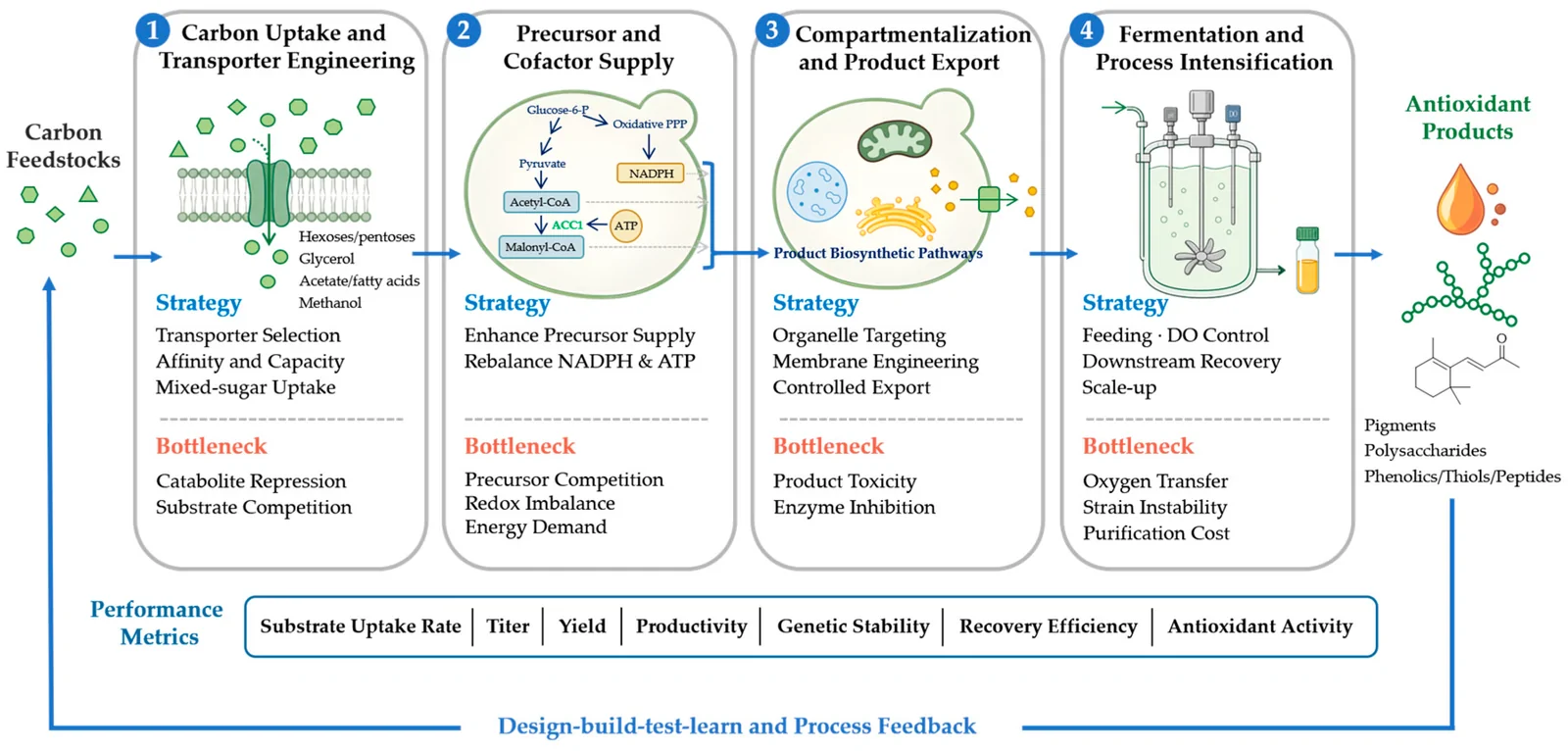
Integrated engineering framework linking carbon uptake to antioxidant biosynthesis and production in yeast. The framework comprises four interconnected modules: (**1**) carbon uptake and transporter engineering; (**2**) intracellular precursor and cofactor supply through glycolysis, the oxidative pentose phosphate pathway, cytosolic acetyl-CoA and malonyl-CoA formation, and NADPH and ATP balancing; (**3**) pathway compartmentalization, intracellular storage, secretion, and product export; and (**4**) fermentation control, process intensification, and product recovery. Representative engineering strategies and major bottlenecks are summarized within each module. Process performance is evaluated using substrate uptake rate, product titer, yield, productivity, genetic stability, recovery efficiency, and antioxidant activity. Blue arrows indicate the progression between engineering levels and the design–build–test–learn feedback loop, green arrows represent substrate or product transport, and orange symbols represent antioxidant products. The figure was created using Microsoft PowerPoint.

The engineering strategies discussed below can be organized into four interconnected levels: carbon uptake and transporter engineering, intracellular precursor and cofactor supply, compartmentalization and product export, and fermentation-process optimization. As summarized in Figure 3, these levels collectively connect carbon feedstock utilization with antioxidant biosynthesis, product accumulation, and process performance.

#### 3.4.1. Substrate Access and Transporter Engineering

Substrate-uptake engineering aims to match carbon entry with the metabolic capacity and precursor requirements of the production host. Relevant targets include transporter abundance, substrate affinity and specificity, and competition among sugars in mixed feedstocks. For example, mutagenesis of Gal2p improved pentose utilization and partially reduced glucose preference in engineered *S. cerevisiae* [107]. Such improvements establish opportunities for expanding substrate use, but their benefits for antioxidant production require evaluation in the corresponding production strains.

Transport engineering can also support the uptake of supplementary precursors. In resveratrol-producing *Y. lipolytica*, introduction of a malonate transporter was combined with malonyl-CoA-forming modules to improve precursor availability [9]. Because uptake and intracellular conversion were engineered together, the resulting production increase reflects the combined intervention. Transporter selection should therefore be tested with the intended feedstock composition and substrate concentrations, using uptake rates, antioxidant yield, biomass formation, and by-product profiles to determine whether increased carbon entry is productively utilized.

#### 3.4.2. Precursor, Cofactor, and Pathway Engineering

Once substrate access is sufficient, intracellular carbon allocation should be adjusted to meet the precursor and cofactor requirements of the target antioxidant. Coordinated enhancement of cytosolic acetyl-CoA and NADPH supply improved β-carotene production in *Y. lipolytica* [8]. For phenolic acids, engineering the supply and recycling of FADH_2_, *S*-adenosyl-L-methionine, and NADPH demonstrated the importance of balancing multiple cofactor systems rather than treating reducing power as an isolated target [10].

Pathway enzyme capacity and regulation can impose additional constraints. Relieving lycopene-induced substrate inhibition of lycopene cyclase improved carotenoid production in *Y. lipolytica* [148]. In canthaxanthin-producing *S. cerevisiae*, screening β-carotene ketolase CrtW variants and deleting *GAL80*, which encodes a repressor of Gal4-mediated transcription, were combined with pathway optimization to support production from sucrose [43]. For ergothioneine production in *Y. lipolytica*, fusion of the *Trichoderma reesei* enzymes TrEgt1 and TrEgt2 was combined with multicopy integration and precursor-supply engineering [77]. These strategies illustrate the need to coordinate precursor formation, cofactor regeneration, enzyme activity, and expression level, because strengthening one component can shift the limitation to another.

#### 3.4.3. Compartmentalization and Product Export

Subcellular localization can influence antioxidant production by altering enzyme access to intermediates, separating competing reactions, and providing suitable environments for product accumulation. Temporospatial pathway control improved heterologous lutein production in *S. cerevisiae* [41]. In *Y. lipolytica*, astaxanthin-pathway targeting was evaluated in lipid bodies, the endoplasmic reticulum, and peroxisomes, individually and in combination, demonstrating the potential of compartment-specific pathway organization to improve production [149]. Compartment selection should therefore consider both biosynthetic activity and the capacity to accommodate the resulting product.

Product export provides a complementary strategy for managing intracellular accumulation. In β-carotene-producing *S. cerevisiae*, overexpression of *SNQ2*, encoding the endogenous ATP-binding cassette transporter Snq2p, was combined with enhanced ATP supply and overexpression of *OLE1*, encoding a Δ^9^ fatty acid desaturase. The resulting changes in energy availability and membrane fluidity improved β-carotene secretion and intracellular production in a two-phase fermentation system [150].

Product accumulation can also be improved by addressing intracellular handling and degradation. In anthocyanin-producing *S. cerevisiae*, expression of the apple glutathione *S*-transferase MdGSTF6 together with deletion of *EXG1*, encoding an exo-1,3-β-glucanase implicated in anthocyanin degradation, improved anthocyanin accumulation [54]. These interventions address product accumulation and stability, which are distinct from export across the plasma membrane. Evaluating compartmentalization and export therefore requires measurements of intracellular and extracellular product fractions, total product yield, and cell growth.

#### 3.4.4. Fermentation Control and Evidence Priorities

Fermentation performance depends on whether engineered production capacity can be maintained without excessive physiological burden. Strong heterologous expression and membrane-protein overproduction can increase resource demands and compromise growth. In β-carotene-producing *S. cerevisiae*, *SNQ2* overexpression reduced intracellular ATP by 85.7% at 36 h and final biomass by 27.9% relative to the control strain [150]. Such trade-offs motivate evaluation of controlled gene copy numbers and dynamic or growth-decoupled expressions. Production stability should also be assessed during repeated cultivation without selection, measuring both genotype retention and product titer to detect the emergence of lower-producing variants.

Oxygen availability, feeding strategy, and substrate concentration determine how effectively the engineered pathway operates during cultivation. Dissolved-oxygen-feedback-controlled (DO-stat) fed-batch cultivation achieved a β-carotene titer of 2.01 g/L in engineered *Y. lipolytica* [151]. However, laboratory agitation and aeration settings cannot be transferred directly to larger vessels. Scale-up must account for the volumetric oxygen transfer coefficient *k*_L_*a*, mixing time, heat removal, foam formation, and substrate gradients. Uptake rates, oxygen demand, biomass formation, and product titer, yield, and productivity should therefore be evaluated together under conditions representative of the intended production process.

Downstream processing must likewise be matched to the product and its location. Intracellular carotenoids generally require biomass recovery, cell disruption, and extraction, whereas engineered export changes the distribution of the product and the requirements for its recovery [150]. Secreted phenolics and extracellular polysaccharides may reduce the need for cell disruption but still require concentration, fractionation, and removal of medium-derived impurities. Cell disruption, solvent use and recovery, and purification should be included in the downstream cost assessment, particularly when low titers or complex feedstocks increase the volume and impurity load to be processed [18,146]. Product purity, residual solvents, feedstock characteristics, and the properties of the production strain must also meet requirements appropriate to the intended use and jurisdiction. For example, the European Food Safety Authority guidance for novel foods requires information on product identity, production process, composition, specifications, proposed uses, and safety; a history of use of the host does not by itself establish the safety of every derived product [152]. Process evaluation should consequently include genetic stability, recovery efficiency, and product quality alongside fermentation performance.

**Table 3:** Representative studies linking carbon-source utilization and engineering interventions to antioxidant production in yeasts.

Product	Yeast Host	Carbon Source	Engineering or Process Intervention	Product Titer	Cultivation Mode	Reference
β-Carotene	*S. cerevisiae*	Xylose	β-Carotene pathway; xylose-associated respiratory and acetyl-CoA phenotype	772.8 mg/L	Fed-batch bioreactor	[147]
β-Carotene	*Y. lipolytica*	Glucose	Cytosolic acetyl-CoA, PPP-derived NADPH, and lipid-flux rebalancing	809.2 mg/L	Shake flask	[8]
β-Carotene	*Y. lipolytica*	Glucose	Lycopene-cyclase engineering; MVA/IUP augmentation; storage balance	39.5 g/L	3 L fed-batch	[148]
β-Carotene	*Y. lipolytica*	Glucose	DO-stat feeding; respiratory and redox control	2.01 g/L	DO-stat fed-batch	[151]
β-Carotene	*S. cerevisiae*	Glucose + acetate	*SNQ2* overexpression; ATP supply; *OLE1* overexpression; dodecane overlay	149.8 mg/L intracellular; 10.1 mg/L secreted	Shake flask	[150]
Resveratrol	*Y. lipolytica*	Glucose + malonate	Heterologous pathway; malonyl-CoA modules; malonate transporter	51.8 mg/L	Shake flask	[9]
Caffeic/Ferulic acids	*S. cerevisiae*	Glucose	FADH_2_, SAM, and NADPH supply and recycling	5.5 ± 0.2/3.8 ± 0.3 g/L	Fed-batch	[10]
Anthocyanins	*S. cerevisiae*	Glucose	MdGSTF6 expression; pathway-enzyme screening; *EXG1* deletion	15.1 mg/L	5 L fed-batch bioreactor	[54]
Canthaxanthin	*S. cerevisiae*	Sucrose	CrtW screening; *GAL80* deletion; carotenoid-pathway optimization	60.36 ± 1.51 mg/L	5 L fed-batch bioreactor	[43]
Ergothioneine	*Y. lipolytica*	Glucose	TrEgt1–TrEgt2 fusion and multicopy integration; precursor-flux redirection	8.03 g/L	5 L fed-batch	[77]

Values and cultivation modes are reported as described in the cited studies and are not directly comparable across products. Abbreviations and gene/protein symbols: CrtW, β-carotene ketolase; DO-stat, dissolved-oxygen-based feedback control; *EXG1*, gene encoding exo-1,3-β-glucanase; FADH_2_, reduced flavin adenine dinucleotide; *GAL80*, gene encoding a repressor of Gal4-mediated transcription; IUP, isopentenol utilization pathway; MdGSTF6, *Malus domestica* glutathione *S*-transferase F6 associated with anthocyanin accumulation; MVA, mevalonate pathway; NADPH, reduced nicotinamide adenine dinucleotide phosphate; *OLE1*, gene encoding Δ^9^ fatty acid desaturase; PPP, pentose phosphate pathway; SAM, S-adenosyl-L-methionine; *SNQ2*, gene encoding the ATP-binding cassette multidrug efflux transporter Snq2p; TrEgt1, bifunctional histidine methyltransferase/sulfoxide synthase from *Trichoderma reesei* involved in ergothioneine biosynthesis; TrEgt2, pyridoxal phosphate-dependent C–S lyase from *T. reesei* involved in ergothioneine biosynthesis.

## 4. Discussion and Perspective

Most production improvements reported to date have resulted from combined interventions that address transport, precursor supply, cofactor balance, pathway capacity, product storage or export, and fermentation control [8,9,10,147,148,149,150,151]. Consequently, the contribution of an individual transporter is often difficult to disentangle from downstream metabolic effects. Future studies should combine defined transporter perturbations with measurements of substrate-uptake rates, antioxidant titers, yields, productivities, biomass formation, and by-product accumulation under matched conditions. Greater standardization of product identification and antioxidant assays is also needed. For industrial application, genetic stability, feedstock variability, inhibitory compounds, oxygen-transfer limitations, product recovery, purity, and regulatory requirements should be evaluated alongside fermentation performance [152].

The evidence reviewed here supports the central premise that carbon-source transport can influence antioxidant production in yeast, but the magnitude of this influence dependents on the substrate, host, and target product. For native and heterologously synthesized intracellular antioxidants, carbon uptake may affect precursor formation, energy generation, and cofactor availability. Cell-wall and extracellular polysaccharides are additionally constrained by sugar-nucleotide formation, polymerization, and secretion, whereas fermentation-, extraction-, and mixed-culture-associated products cannot always be directly linked to yeast carbon metabolism. Therefore, increased substrate uptake should not be interpreted as enhanced antioxidant biosynthesis unless the subsequent allocation of the imported carbon is also demonstrated.

## 5. Conclusions

Yeasts are both natural sources and versatile production hosts for antioxidants, but the biological origin and processing history of each product must be considered when relating its formation to yeast metabolism. Carbon-source transport represents a product- and substrate-dependent control point rather than a universal production bottleneck. Effective improvement therefore requires coordinated optimization of substrate uptake, intracellular carbon allocation, precursor and cofactor supply, pathway activity, product accumulation or export, and fermentation conditions. This integrated framework can guide the selection of suitable yeast hosts, feedstocks, and engineering strategies for antioxidant production.

## Data Availability

No new data were created or analyzed in this study. Data sharing is not applicable to this article.
